# Three-dimensional CT imaging features of impacted first permanent molars and their correlation with prognosis

**DOI:** 10.1007/s11282-026-00933-7

**Published:** 2026-05-16

**Authors:** Jong Woo Kim, Kyung-Hoe Huh, Won-Jin Yi, Min-Suk Heo, Sam-Sun Lee, Jo-Eun Kim

**Affiliations:** 1 Department of Oral and Maxillofacial Radiology, Seoul National University Dental Hospital, Seoul, 03080 Republic of Korea; 2 Department of Oral and Maxillofacial Radiology, School of Dentistry and Dental Research Institute, Seoul National University, Seoul, 03080 Republic of Korea

**Keywords:** Tooth, Impacted, Root resorption, Tomography, X-ray computed, Prognosis

## Abstract

**Objectives:**

Impaction of first permanent molars is uncommon but may affect oral function and craniofacial development. Although various etiologic factors have been proposed, three-dimensional imaging features associated with impacted first permanent molars—particularly, furcation area root resorption—have not been well investigated. The objective of this study is to analyze CT imaging features of impacted first permanent molars and determine their correlation with prognosis.

**Methods:**

This retrospective cohort study reviewed multidetector computed tomography and cone-beam computed tomography scans of 71 patients diagnosed with impaction of first permanent molars. Two oral and maxillofacial radiologists evaluated CT imaging features such as peri-coronal disturbance, root dilaceration, apical closure, ankylosis, and furcation area root resorption. Treatment methods and eruption outcomes were also recorded. Logistic regression analysis was performed to identify imaging features associated with eruption failure of impacted first permanent molars.

**Results:**

Among 71 impacted first molars, peri-coronal disturbance was observed in 29.6%, root dilaceration in 31.0%, apical closure in 42.3%, ankylosis in 11.3%, and furcation area root resorption in 35.2% of cases. After treatment, furcation area root resorption showed 64.7% (11/17) of eruption failure. Notably, furcation area root resorption was identified as the only significant imaging feature associated with eruption failure (OR 8.32; 95% CI 1.15–60.31; *p* = 0.036).

**Conclusions:**

Furcation area root resorption is a relatively common CT imaging feature in impacted first permanent molars and is associated with poor prognosis. Because this feature is often undetectable on plain radiographs, 3D CT evaluation should be considered for accurate diagnosis and appropriate treatment planning.

## Introduction

A tooth is considered impacted if it fails to erupt into its normal functional position within the expected developmental time frame [1]. Impacted permanent teeth are most commonly seen in maxillary and mandibular third molars, followed by mandibular and maxillary premolars and mandibular canines [2]. The reported prevalence of permanent molar impaction varies depending on the study, population, and specific molar type. First permanent molars show a lower impaction rate than second molars [3]. Most studies demonstrate a low prevalence of 0.01% for both maxillary and mandibular first molars [4, 5], although some studies report rates as high as 0.4% [6].

Impaction of permanent molars can have significant consequences for oral function and craniofacial development. The absence of permanent molars compromises masticatory efficiency and occlusal support, and may hinder normal tooth eruption and alveolar process formation, ultimately contributing to reduced facial height [7]. In addition, such impaction often induces secondary changes, including mesial tipping of adjacent teeth, asymmetrical molar relationships, midline deviation toward the affected side, and overeruption of opposing teeth, resulting in malocclusion [8–11].

The main cause of impaction of permanent molars is the presence of a physical obstacle or an abnormal inclination of the eruption tooth [2]. The impaction arises from eruptive obstructions, including supernumerary teeth, odontomas, cysts or other space-occupying lesions within the eruption pathway [8, 12]. Ectopic eruption of permanent molars can lead to their impaction, often against other teeth [13]. Moreover, previous studies using two-dimensional radiographs demonstrated that root anomalies, such as abnormal crown-to-root ratios, root dilaceration, and shortened mesial roots relative to distal roots, were associated with the impaction of mandibular second permanent molars [14, 15].

Management strategies for impacted permanent molars range from removal of the obstruction and excision of the overlying tissue to orthodontic or surgical uprighting and extraction [2]. The existing literature consistently demonstrates that resolving the underlying obstruction or facilitating the proper eruption pathway is associated with favorable clinical outcomes [16].

In the interpretation of CT scans for impacted permanent molars, root resorption at the furcation area is a frequent clinical observation; however, it has seldom been documented in previous studies [17]. This clinical finding raises a critical question about whether furcation area root resorption is a *cause* or a *consequence* of molar impaction. Consequently, it is necessary to investigate impacted molars, particularly, the first molars, by analyzing their 3D imaging features. The objective of the present study, therefore, is to comprehensively analyze various CT features observed in a relatively large sample of impacted first permanent molars and identify imaging-based predictors for their prognosis.

## Materials and methods

### Patient selection

This study was approved by the Institutional Review Board (IRB No. ERI25064). The requirement of patient consent was waived due to the retrospective nature of the study. We retrospectively reviewed MDCT or CBCT data of patients who visited a tertiary dental hospital between January 2013 and December 2024. We used the keywords “impacted tooth”, “submerged tooth”, “delayed eruption”, “impaction” or “ankylosis” in the search within the report text section of radiology reports using the Picture Archiving and Communications System (INFINITT PACS, INFINITT Healthcare, Seoul, South Korea). Only patients with impacted first permanent molars were included in the study. A total of 85 patients with impacted first permanent molars were identified. After excluding those without follow-up records or still under follow-up, imaging analysis was conducted on 71 patients.

### Image analysis

From the dataset, 71 CT images (4 MDCT, 67 CBCT images) were analyzed. CBCT images were obtained using a CS9300 unit (Carestream Health, Rochester, NY, USA) with the following field of view (FOV) settings and corresponding voxel sizes: 5 × 5 cm (90 μm), 10 × 5 cm (180 μm), and 17 × 6 cm (200 μm). MDCT images were obtained using a SOMATOM Definition Edge scanner (Siemens Healthineers, Forchheim, Germany) with a matrix size of 512 × 512. Although variations in FOV in CBCT were present, all images provided sufficient coverage of the impacted first permanent molars and adjacent anatomical structures, and no cases were excluded due to inadequate visualization. Radiological evaluations were independently performed by two oral and maxillofacial radiologists with clinical experience of 5 and 15 years, respectively. In cases of disagreement, the images were re-evaluated jointly and a final decision was reached by consensus.

**Fig. 1:**
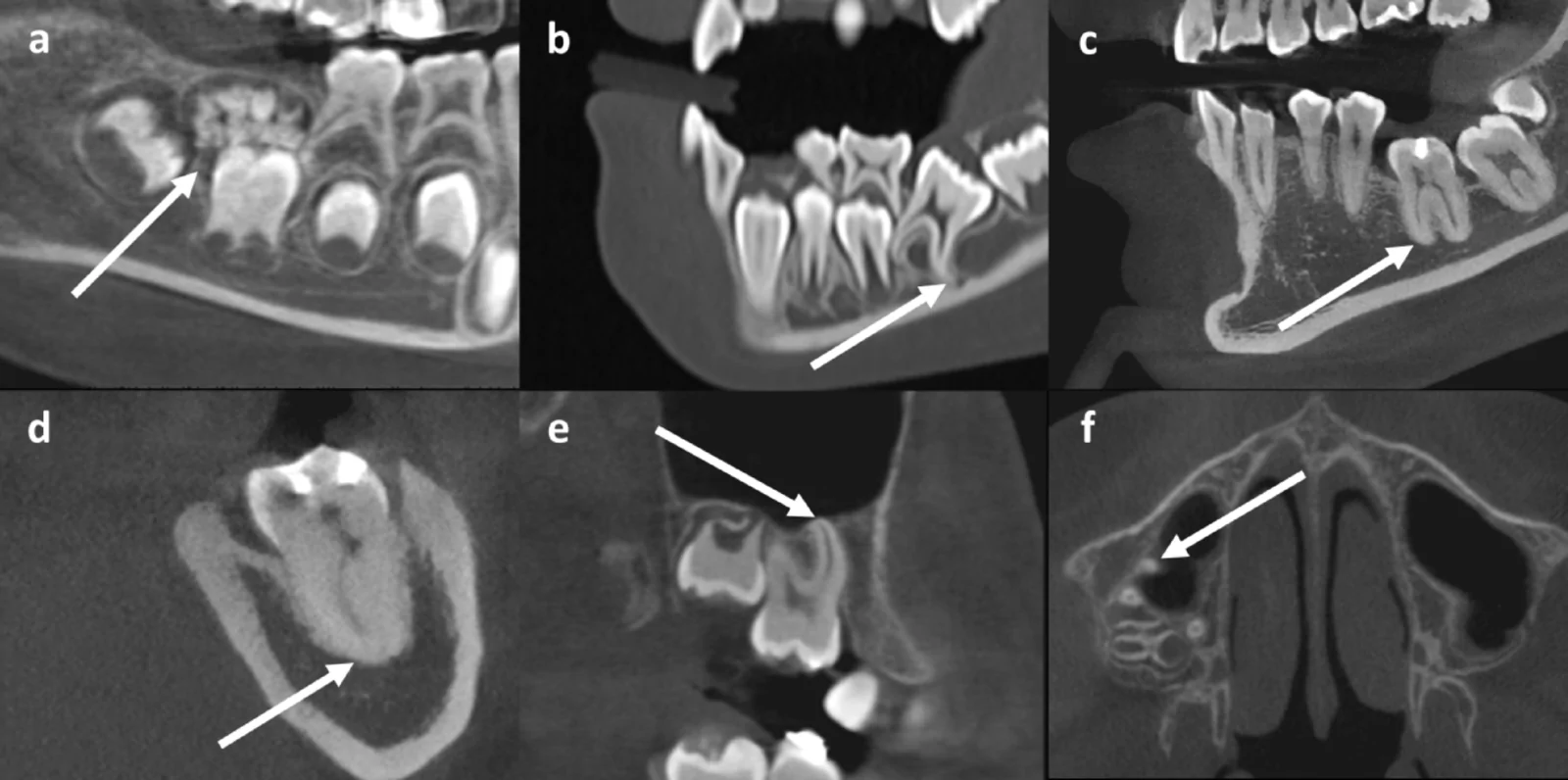
Representative CT imaging features of impacted first permanent molars. **a** Cropped sagittal view showing peri-coronal disturbance. **b** Cropped sagittal view showing root dilaceration. **c**, **d** Cropped sagittal and coronal views showing ankylosis. **e**, **f** Cropped sagittal and axial views showing apical closure. Arrows indicate each feature

**Fig. 2:**
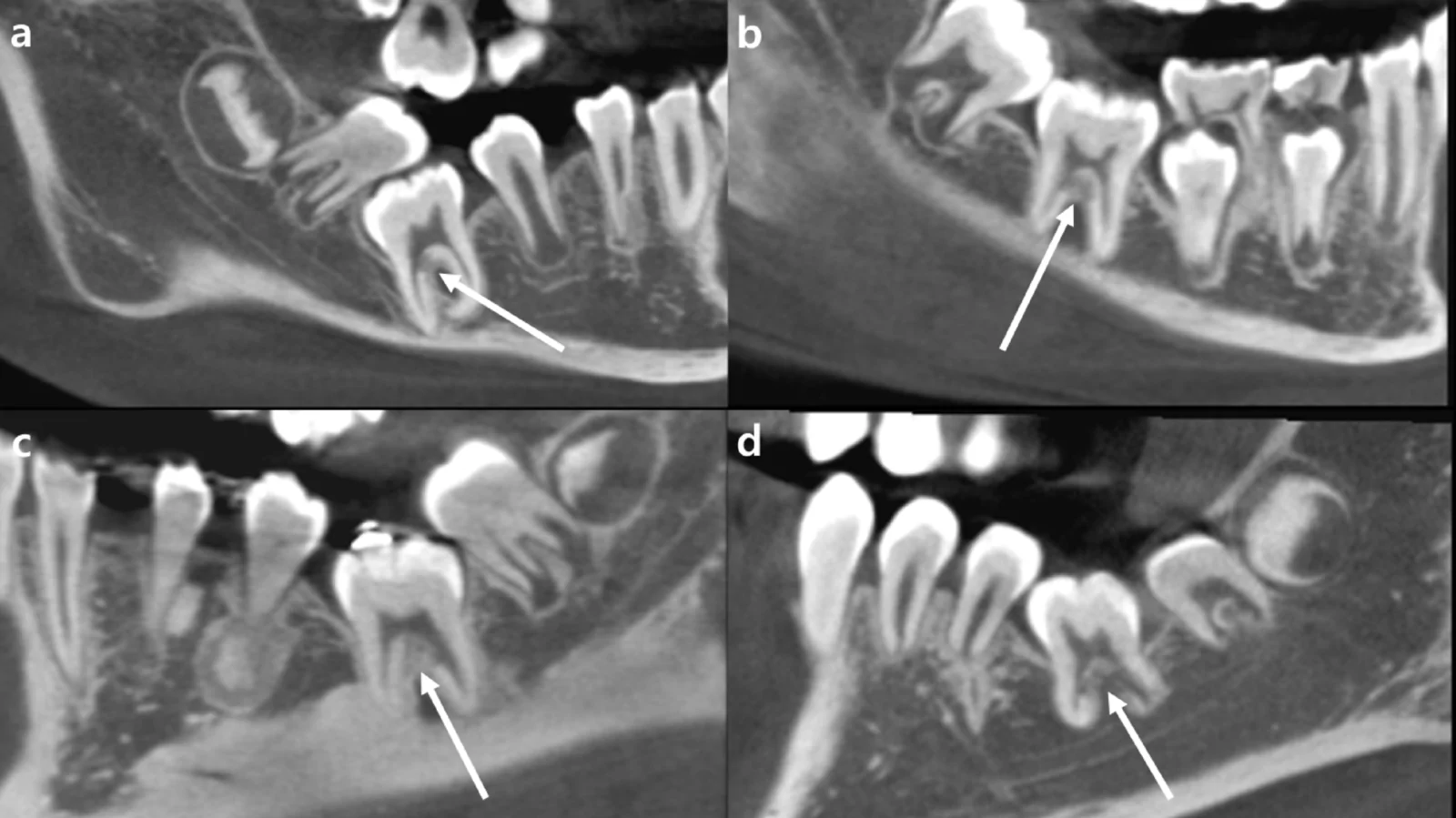
Representative CT images of furcation area root resorption. **a**, **b** Cropped sagittal views showing furcation area root resorption without bony replacement. **c**, **d** Cropped sagittal views showing furcation area root resorption with bony replacement. Arrows indicate the site of furcation area root resorption

Peri-coronal disturbance, root dilaceration, apical closure, ankylosis and furcation area root resorption were analyzed by observing each CT image in axial, coronal and sagittal planes. First, peri-coronal disturbances were defined as obstacles located above or around the crowns of the impacted teeth that interfered with their eruption. These obstacles include odontogenic benign tumors (such as odontoma, ameloblastic fibroma, and ameloblastic fibro-odontoma), hyperplastic follicles with calcification, and mesial locking by a deciduous tooth (Fig. 1a). Second, root dilaceration was defined as an abrupt change in root angulation that could potentially affect eruption or extraction (Fig. 1b). Third, ankylosis was defined as a direct contact between the root and the alveolar bone without the presence of a periodontal ligament (PDL) space (Fig. 1c and d). Fourth, apical closure was defined as the tapering of the root canal toward the apex, which appeared closed on sagittal and axial CT images (Fig. 1e, f). The developmental stage expected for the patient’s age was also taken into account. Lastly, furcation area root resorption was defined as the localized irregular loss of dentin at the furcation area resulting in a decrease in density or replacement by alveolar bone compared to adjacent normal dentin, when observed on three orthogonal planes of the CT images, especially on a sagittal plane (Fig. 2). For furcation area root resorption, it was determined whether it was possible to detect it on panoramic radiographs.

### Treatment and prognosis evaluation

The treatments conducted to resolve the impacted permanent first molars were examined from electronic dental records. Whether the impacted teeth were erupted or extracted was assessed through follow-up records and images. The period of total follow-up was also recorded.

### Statistical analysis

Descriptive statistics were conducted to summarize the demographic and imaging characteristics of the study population. Continuous variables such as age and follow-up period were presented as means and standard deviations. Categorical variables, including gender, tooth position, and radiographic findings (peri-coronal disturbance, root dilaceration, apical closure, ankylosis, furcation area root resorption, and detectability on panoramic radiographs), were summarized as frequencies and percentages. In addition, age-related trends were evaluated by comparing the mean age between groups with and without each imaging feature using Welch’s t test. Logistic regression analysis was performed to identify imaging features associated with the eruption failure of impacted first permanent molars. All statistical analyses were performed using Python (version 3.11). A p value less than 0.05 was considered statistically significant.

## Results

A total of 71 patients were included in the study, comprising 41 males (57.7%) and 30 females (42.3%). The most frequently impacted teeth were mandibular right first molar (31/71, 43.7%) followed by mandibular left first molar (21/71, 29.6%), maxillary right first molar (12/71, 16.9%) and maxillary left first molar teeth (7/71, 9.9%). The mean age of the patients at diagnosis was 10.35 ± 5.44 years (range 5–41 years), and the mean follow-up period was 20.54 ± 20.69 months (range 1–123 months). All patients were referred from local clinics for delayed eruption or impaction of the first permanent molars and presented no associated symptoms. Except one patient with craniosynostosis, no significant findings related to past medical history were identified.

Regarding CT imaging features, peri-coronal disturbance was observed in 29.6% of patients, root dilaceration in 31.0%, apical closure in 42.3%, ankylosis in 11.3%, furcation area root resorption in 35.2% and non-specific findings in 23.9% (Table 1). In addition, among cases with furcation area root resorption identified on CT, only 44% (11/25) were detectable on panoramic radiographs.

**Table 1:** Demographic information and CT imaging features

	Number	Ratio
Age (years)	10.35 ± 5.44	
*Sex*		
Male	41	57.7%
Female	30	42.3%
*Location*		
#16	12	16.9%
#26	7	9.9%
#36	31	43.7%
#46	21	29.6%
*Imaging feature*		
Peri-coronal disturbance	21	29.6%
Root dilaceration	22	31.0%
Apical closure	30	42.3%
Ankylosis	8	11.3%
Furcation area root resorption	25	35.2%
Non-specific	17	23.9%

Imaging features can appear in multiple forms in a single impacted tooth

**Fig. 3:**
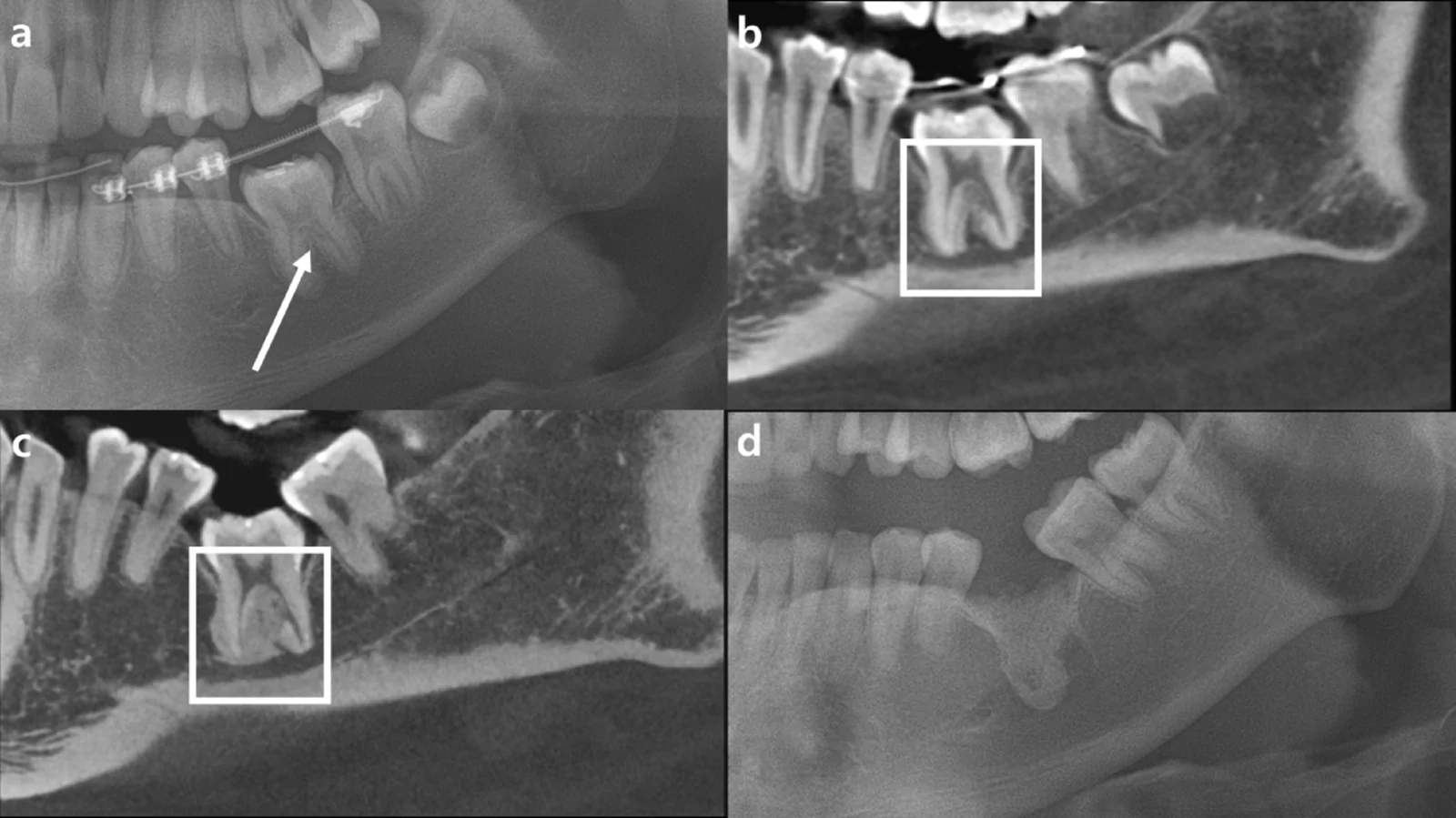
A case of treatment failure in an impacted first permanent molar with furcation area root resorption. **a** Cropped panoramic image taken after gingivectomy. The arrow indicates the lesion of furcation area root resorption. **b** Cropped sagittal CT view taken during forced eruption. **c** Cropped sagittal CT view taken 5 years after treatment. **d** Cropped panoramic image taken after the extraction of the tooth (6 years post-treatment). Note the progression of furcation area root resorption on #36 (**b**, **c**, white boxes)

Treatment methods and post-treatment eruption outcomes were summarized according to CT imaging features in Table 2. When a disturbance was present, surgical removal of the obstacle generally resulted in successful eruption (19/20, 95%). In contrast, cases with furcation area root resorption frequently failed to erupt despite interventions such as forced eruption (11/17, 64.7%; Fig. 3). Furthermore, when ankylosis was clearly identified on imaging, treatment was rarely attempted.

**Table 2:** Summary of eruption outcomes after treatment based on CT imaging features

Imaging feature	Treatment status	Treatment type	Prognosis
Peri-coronal disturbance (21)	Treated (20)	Forced eruption + surgical excision (16)	Eruption
		Forced eruption (1)	Eruption
		Surgical excision (2)	Eruption
		Forced eruption (1)	Extraction (fail)
	Untreated (1)	–	Extraction (fail)
Root dilaceration (22)	Treated (15)	Forced eruption (8)	Eruption
		Intentional luxation + surgical repositioning (1)	Eruption
		Forced eruption + surgical excision (6)	Extraction (fail)
	Untreated (7)	–	Extraction (fail)
Apical closure (30)	Treated (18)	Forced eruption (3)	Eruption
		Forced eruption + surgical excision (1)	Eruption
		Intentional luxation + surgical repositioning (3)	Eruption
		Surgical excision (1)	Eruption
		Forced eruption + surgical excision (6)	Extraction (fail)
		Forced eruption + intentional luxation (4)	Extraction (fail)
	Untreated (12)	–	Extraction (fail)
Ankylosis (8)	Treated (1)	Forced eruption (1)	Extraction (fail)
	Untreated (7)	–	Extraction (fail)
Furcation area root resorption (25)	Treated (17)	Forced eruption (2)	Eruption
		Surgical excision (1)	Eruption
		Intentional luxation + surgical repositioning (3)	Eruption
		Forced eruption + surgical excision (10)	Extraction (fail)
		Intentional luxation (1)	Extraction (fail)
	Untreated (8)	–	Extraction (fail)
Non-specific (17)	Treated (16)	Forced eruption (2)	Eruption
		Forced eruption + surgical excision (5)	Eruption
		Surgical excision (8)	Eruption
		Intentional luxation (1)	Extraction (fail)
	Untreated (1)	–	Extraction (fail)

Numbers in parentheses indicate the number of patients

Statistical analysis (Welch’s t test) revealed significant age-related trends for several CT imaging features. The mean age of patients with furcation area root resorption was significantly higher than that of those without the feature (12.20 years vs. 9.35 years; *p* = 0.016). Similarly, ankylosis (20.62 years vs. 9.05 years; *p* = 0.013) and apical closure (13.87 years vs. 7.78 years; *p* < 0.001) were significantly associated with higher mean age. In contrast, peri-coronal disturbance was significantly associated with lower mean age (8.48 years vs. 11.14 years; *p* = 0.023). Root dilaceration did not show a statistically significant age-related trend (12.27 years vs. 9.49 years; *p* = 0.12).

Logistic regression analysis identified furcation area root resorption as the sole significant independent predictor of eruption failure (Odds Ratio [OR] = 8.32, 95% confidence interval [CI] = 1.15–60.31, *p* = 0.036). Accordingly, impacted first permanent molars with furcation area root resorption exhibited a significantly lower probability of successful eruption. Other variables, including peri-coronal disturbance, root dilaceration, apical closure, ankylosis, and age, showed no significant association with eruption outcomes (Table 3).

**Table 3:** Logistic regression analysis of CT imaging features and age associated with eruption failure

Imaging feature	OR	95% CI	*p* value
Peri-coronal disturbance	~ 0.00	~ 0–∞	1.000
Root dilaceration	0.52	0.07–3.82	0.520
Apical closure	5.99	0.65–55.31	0.114
Ankylosis	~ 6.72e^20^	~ 0–∞	1.000
Furcation area root resorption	8.32	1.15–60.31	0.036
Age	0.84	0.57–1.22	0.355

*OR* odds ratio, *CI* confidence interval

## Discussion

This study reports the various 3D CT imaging features of the impacted first permanent molars, including the cases of furcation area root resorption. To the best of our knowledge, the correlation between the impaction of the first permanent molars and their prognosis based on the 3D imaging features, especially furcation area root resorption, has not been widely studied. Our findings indicate that the presence of furcation area root resorption was significantly associated with eruption failure.

In this study, peri-coronal disturbance was observed in 29.6% (21/71) of impacted first permanent molars and 95% (19/20) of the impacted first permanent molars with peri-coronal disturbance were erupted after the treatment. According to the study by la Monaca et al. [16], the treatment outcome for the removal of pathologic conditions such as odontomas and supernumerary teeth were excellent (18/18 molars), which is in line with our study. Root dilaceration was observed in 31.0% (22/71) of impacted first permanent molars and 60% (9/15) of the impacted first permanent molars with root dilaceration were erupted after the treatment according to our results. Irrespective of whether treatment was performed, the overall rate of eruption failure was 59.1% (13/22). The study by Palma et al. [9] showed that root dilaceration was observed in 25.7% (9/35) of impacted first and second molars and that 88.9% (8/9) of the impacted molars with root dilaceration failed to erupt. However, it is worth noting that their sample size was limited compared to ours and the treatment status of the included teeth was not specified. Thus, despite differences in sample size and treatment protocols between the studies, the overall rate of eruption failure for root dilaceration in the present study is largely consistent with the previous findings. Palma et al. [9] further noted that the presence of root dilaceration should not be considered an etiologic cause, but rather a consequence responsible for the lack of space needed for eruption. Our results support this interpretation as 9 out of 15 treated molars with root dilaceration erupted successfully (8 via forced eruption), suggesting that the underlying cause of impaction may be insufficient space rather than the root dilaceration itself.

Apical closure was the most prevalent CT imaging feature, accounting for 42.3% of the study population. However, given the absence of literature suggesting a causal relationship between apical closure and impaction of permanent molars, it is improbable that this feature serves as an etiological factor. Rather, it likely reflects the patients’ dental age at the time of diagnosis, aligning with findings by Palma et al. [9], who reported that 80% of impacted permanent molars were in the late stages of root development. Moreover, according to our results, 55.6% (10/18) of the impacted first permanent molars with apical closure failed to erupt after the treatment. This failure may be attributed to the advanced stage of root development, where the natural eruptive force is less vigorous than in earlier stages; such diminished eruptive potential in mature teeth appears to have compromised the response to the treatment.

The statistical analysis of age-related trends showed that furcation area root resorption was significantly associated with older age (*p* = 0.016), which suggests that the prevalence or detection of this resorption may increase as the tooth remains impacted over a longer period. Peri-coronal disturbance was significantly associated with younger patients (*p* = 0.023), which is consistent with the fact that these disturbances often lead to early-stage impaction. Both apical closure (*p* < 0.001) and ankylosis (*p* = 0.013) were significantly associated with older patients, reflecting the stage of root development.

Regarding furcation area root resorption, which represents the key finding in our study, few studies have specifically associated this finding with either the etiology or the consequence of tooth impaction. According to the study by Heithersay [18], maxillary central incisors were most commonly affected by cervical root resorption, followed by maxillary canines, maxillary lateral incisors, mandibular first molars and maxillary first molars, all of which were analyzed on plain radiographs. In the same vein, the study by Patel et al. [19], which investigated all external cervical resorption cases by using both periapical radiographs and CBCT, found that maxillary central incisors (35/115, 30.4%) were the most commonly affected, followed by mandibular first molars (18/115, 15.7%), mandibular central incisors (13/115, 11.3%), and maxillary molars (12/115, 10.4%). It should be noted that the existing literature has primarily focused on cervical root resorption, whereas root resorption occurring in the furcation area has been rarely addressed. In particular, no previous study has examined furcation area root resorption as a 3D imaging feature in the context of impacted first molars, which makes our study meaningful in that it highlights furcation area root resorption as a distinct imaging feature and suggests its potential clinical relevance. This is noteworthy because many clinicians are unaware that this finding is often found in impacted first permanent molars. In our study, 35.2% (25/71) of the impacted first molars exhibited furcation area root resorption, which was the second most common imaging feature, and that only 35.3% (6/17) of the impacted first molars with furcation area root resorption were erupted after the treatment. It remains unclear whether furcation area root resorption represents a cause or an effect of impaction; however, these results suggest that it is highly probable that furcation area root resorption may be related either to the etiology or consequence of impaction.

The major causes associated with impacted molars are known to include physical obstruction, root anomalies, and idiopathic factors. Accordingly, surgical removal or excision, orthodontic uprighting, and surgical uprighting have been widely employed as treatment approaches to facilitate eruption. Magnusson et al. [20] reported a high success rate for the removal of the overlying tissue, while Fu et al. [21] demonstrated favorable outcomes with orthodontic uprighting in 125 out of 183 mandibular second molars. Furthermore, Caminiti et al. [22] showed outstanding results by treating 260 s mandibular molars with surgical uprighting. Taken together, these findings highlight the importance of early recognition and timely intervention in order to optimize treatment success. For instance, in the present study, when 3D imaging demonstrated loss of PDL space suggestive of ankylosis, unnecessary treatments were avoided. This underscores that identifying the cause is essential for establishing effective treatment plans and emphasizes the critical role of imaging findings. With the increasing routine use of 3D CBCT in diagnosis, the detection of impacted permanent molars has become more frequent. Our findings show that furcation area root resorption of impacted first permanent molars was observed in 25 cases on CT, but the detection rate on plain radiographs was low (11/25, 44%). This indicates the difficulty of identifying such lesions using conventional radiographic methods alone and suggests that meticulous evaluation using CT is necessary.

This study had some limitations. First, although the size of the sample was relatively large compared with previous studies, it was insufficient to draw a definitive statistical conclusion. To exclude other factors related to tooth position, the study was limited to the impaction of the first permanent molar alone, which further restricted the sample size. Second limitation of the study is the imbalance between CBCT and MDCT cases. The majority of datasets consisted of CBCT cases (*n* = 67), while the number of MDCT cases was limited (*n* = 4). Although these modalities differ in spatial resolution, contrast characteristics, and artifact behavior, the imaging features evaluated were primarily based on morphological findings, rather than quantitative measurements. These features can be reliably assessed on both CBCT and MDCT using multiplanar reconstruction. Therefore, the impact of modality differences on the study results is considered limited. Nevertheless, this heterogeneity may limit the generalizability of the findings to MDCT. Third limitation of the study is that as a retrospective cohort study, it relied on chart reviews and existing records, making it difficult to apply a consistent treatment protocol and to accurately analyze prognostic factors. Therefore, further prospective research should be conducted, including second molars and standardized treatment protocols. In addition, genetic and biological analyses should be incorporated to determine whether specific imaging features represent the cause or the result of impaction.

## Conclusions

In conclusion, this study analyzed the 3D CT imaging features and treatment outcomes of impacted first permanent molars. Furcation area root resorption was observed in a substantial proportion of cases, a finding that has been underreported in previous studies. Impacted first permanent molars with furcation area root resorption showed a high rate of eruption failure. Given that furcation area root resorption is associated with poor prognosis and is often difficult to detect on plain radiographs, 3D CT evaluation should be considered for accurate diagnosis and appropriate treatment planning.

## Data Availability

No datasets were generated or analysed during the current study.

## References

[1] American Association of Oral and Maxillofacial Surgeons. Management of impacted third molars: clinical paper. American Association of Oral and Maxillofacial Surgeons; 2024.

[2] Ngan P, Hornbrook R, Weaver B. Early timely management of ectopically erupting maxillary canines. Semin Orthod. 2005;11(3):152–63. 10.1053/j.sodo.2005.04.009.

[3] Aharon ED, Rushinek H, Casap-Caspi N, Chaushu S. Impacted permanent first and second molars and failure of eruption: a comprehensive review for the orthodontic practitioner. Semin Orthod. 2024;30(5):727–41. 10.1053/j.sodo.2024.09.004.

[4] Bereket C, Çakir-Özkan N, Şener I, et al. Retrospective analysis of impacted first and second permanent molars in the Turkish population: a multicenter study. Med Oral Patol Oral Cir Bucal. 2011;16(7):e874–8. 10.4317/medoral.17094. 21196844

[5] Grover PS, Lorton L. The incidence of unerupted permanent teeth and related clinical cases. Oral Surg Oral Med Oral Pathol. 1985;59(4):420–5. 10.1016/0030-4220(85)90070-2. 3858781

[6] Siotou K, Kouskouki MP, Christopoulou I, Tsolakis AI, Tsolakis IA. Frequency and local etiological factors of impaction of permanent teeth among 1400 patients in a Greek population. Dent J (Basel). 2022;10(8):150. 10.3390/dj10080150. PMC9406841 36005248

[7] Proffit WR. Equilibrium theory revisited: factors influencing position of the teeth. Angle Orthod. 1978;48(3):175–86. 10.1043/0003-3219(1978)048<0175:ETRFIP>2.0.CO;2. 280125

[8] Raghoebar GM, Boering G, Vissink A, Stegenga B. Eruption disturbances of permanent molars: a review. J Oral Pathol Med. 1991;20(4):159–66. 10.1111/j.1600-0714.1991.tb00913.x. 2061854

[9] Palma C, Coelho A, González Y, Cahuana A. Failure of eruption of first and second permanent molars. J Clin Pediatr Dent. 2003;27(3):239–46. 10.17796/jcpd.27.3.dm4v13441p161928. 12739684

[10] Chaushu S, Becker A, Chaushu G. Orthosurgical treatment with lingual orthodontics of an infraoccluded maxillary first molar in an adult. Am J Orthod Dentofac Orthop. 2004;125(3):379–87. 10.1016/j.ajodo.2003.05.003. 15014419

[11] Rosner D, Becker A, Casap N, Chaushu S. Orthosurgical treatment including anchorage from a palatal implant to correct an infraoccluded maxillary first molar in a young adult. Am J Orthod Dentofac Orthop. 2010;138(6):804–9. 10.1016/j.ajodo.2008.09.039. 21130340

[12] Bondemark L, Tsiopa J. Prevalence of ectopic eruption, impaction, retention and agenesis of the permanent second molar. Angle Orthod. 2007;77(5):773–8. 10.2319/072506-306.1. 17685771

[13] Raghoebar GM, Boering G, Jansen HW, Vissink A. Secondary retention of permanent molars: a histologic study. J Oral Pathol Med. 1989;18(8):427–31. 10.1111/j.1600-0714.1989.tb01338.x. 2691646

[14] Vedtofte H, Andreasen J, Kjær I. Arrested eruption of the permanent lower second molar. Eur J Orthod. 1999;21(1):31–40. 10.1093/ejo/21.1.31. 10191575

[15] Shapira Y, Finkelstein T, Shpack N, Lai YH, Kuftinec MM, Vardimon A. Mandibular second molar impaction. Part I: genetic traits and characteristics. Am J Orthod Dentofac Orthop. 2011;140(1):32–7. 10.1016/j.ajodo.2009.08.034. 21724084

[16] la Monaca G, Cristalli MP, Pranno N, Galluccio G, Annibali S, Pippi R. First and second permanent molars with failed or delayed eruption: clinical and statistical analyses. Am J Orthod Dentofac Orthop. 2019;156(3):355–64. 10.1016/j.ajodo.2018.09.020. 31474265

[17] Becker A. Orthodontic treatment of impacted teeth. 4th ed. Wiley-Blackwell; 2022.

[18] Heithersay GS. Invasive cervical resorption: an analysis of potential predisposing factors. Quintessence Int. 1999;30(2):83–95. 10356560

[19] Patel K, Mannocci F, Patel S. The assessment and management of external cervical resorption with periapical radiographs and cone-beam computed tomography: a clinical study. J Endod. 2016;42(10):1435–40. 10.1016/j.joen.2016.06.014. 27507628

[20] Magnusson C, Kjellberg H. Impaction and retention of second molars: diagnosis, treatment and outcome: a retrospective follow-up study. Angle Orthod. 2009;79(3):422–27. 10.2319/021908-97.1. 19413377

[21] Fu PS, Wang JC, Wu YM, et al. Impacted mandibular second molars. Angle Orthod. 2012;82(4):670–5. 10.2319/102111-656.1. PMC8845543 22229822

[22] Caminiti MF, El-Rabbany M, Lou T, Reinish EI. Surgical uprighting of mandibular second molars: a single-group retrospective cohort study. Am J Orthod Dentofac Orthop. 2020;158(6):849–55. 10.1016/j.ajodo.2019.11.016. 33131921

